# Visual outcomes after vitrectomy for epiretinal membrane in pseudophakic eyes with a diffractive trifocal intraocular lens: a retrospective cohort study

**DOI:** 10.1186/s12886-022-02273-6

**Published:** 2022-01-27

**Authors:** Luis Arrevola-Velasco, Jaime Beltran, Maria Jesus Gimeno, Julio Ortega-Usobiaga, Vasyl Druchkiv, Fernando Llovet-Osuna, Julio Baviera-Sabater

**Affiliations:** 1 Retina-Vitreous Unit, Refractive Surgery Unit, Clinica Baviera (an AIER EYE HOSPITAL GROUP division), Paseo de la Castellana, 20 28046 Madrid, Spain; 2 Research and Development Clinica Baviera (an AIER EYE HOSPITAL GROUP division), Valencia, Spain; 3 Refractive Surgery Unit, Research and Development, Clinica Baviera (an AIER EYE HOSPITAL GROUP division), Valencia, Spain; 4 Refractive Surgery Unit, Clinica Baviera (an AIER EYE HOSPITAL GROUP division), Bilbao, Spain; 5 Refractive Surgery Unit, Clinica Baviera (an AIER EYE HOSPITAL GROUP division), Madrid, Spain; 6 Refractive Surgery Unit, Clinica Baviera (an AIER EYE HOSPITAL GROUP division), Valencia, Spain

**Keywords:** Diffractive multifocal intraocular lens, Trifocal intraocular lens, Epiretinal membrane, Vitrectomy, Pseudophakic eyes

## Abstract

**Background:**

Diffractive intraocular lenses (IOLs) could affect visual acuity in patients with macular pathologies such as epiretinal membrane (ERM) and could influence the results of pars plana vitrectomy (PPV) for ERM removal in pseudophakic eyes with these IOLs. The aim of this study is to evaluate the effect on visual outcomes of a diffractive trifocal IOL in PPV for ERM peeling.

**Methods:**

This is a retrospective cohort study on 20 eyes with a single model of trifocal IOL that underwent PPV for removal of ERM between January 2015 and September 2018 in our clinics. Follow up was at least 1 year. Primary outcome measure was mean change in visual acuity. Secondary outcome measures were mean change in central macular thickness (CMT), recovery of the external retinal layers, and change in spherical equivalent (SE).

**Results:**

Mean corrected distance visual acuity (CDVA) was 0.03 ± 0.03 logMAR after phacoemulsification; this worsened to 0.23 ± 0.10 logMAR with ERM, improving to 0.10 ± 0.04 log MAR 12 months after PPV (*p* = 0.001). Mean uncorrected near visual acuity (UNVA) was Jaeger 2.62 ± 0.51 after lensectomy. This worsened to Jaeger 5.46 ± 1.67 with ERM and improved to the initial Jaeger 2.69 ± 0.84 after PPV (*p* = 0.005). CMT decreased significantly, from 380.15 ± 60.50 μm with the ERM to 313.70 ± 36.98 μm after PPV. Mean SE after lensectomy was − 0.18 ± 0.38 D, which minimally changed to – 0.18 ± 0.47 D after PPV (*p* = 0.99). The only complication recorded after PPV was a case of cystoid macular edema. No difficulties in visualization due to IOL design were reported during PPV.

**Conclusion:**

PPV for ERM in eyes with this trifocal IOL seems to be safe and effective, and allows recovery of the loss of UNVA.

## Background

Epiretinal membrane (ERM) is a pathological proliferation of fibrotic and glial tissue over the internal limiting membrane (ILM) of the macula. It can be idiopathic or secondary to retinal conditions such as trauma, retinal detachment, and vascular or inflammatory diseases [1]. The prevalence of ERM increases from < 1% in patients aged under 50 years to between 15 and 28.1% in patients aged over 65 years, with no differences between sex [2]. ERM is more frequent after posterior vitreous detachment [2, 3] and may produce visual impairment and metamorphopsia. The only treatment option is pars plana vitrectomy (PPV) and ERM peeling, which was first described by Machemer in 1978 [4]. Good visual and anatomical results have been reported since the introduction of the 23G and 25G microincision techniques [5]. The most important factors for visual prognosis are those related to distortion of the retinal layers, especially the outer segments, central macular thickness (CMT) and inner retinal layer thickening and migration, as determined by spectral domain optical coherence tomography (SD-OCT) [6, 7].

According to Eurostat, more than 4.3 million lensectomies were performed in the European Union in 2018, and a multifocal intraocular lens (IOL) was implanted in 5–10% of them depending on the country [8]. These procedures involved both cataract surgeries and refractive surgeries in clear lenses, both of which have proven very successful, even after long-term follow-up. Consequently multifocal IOLs are being implanted in younger patients [9]. A reduction in contrast sensitivity has been reported with diffractive trifocal IOLs [10] and many authors therefore recommend avoiding implantation of a multifocal IOL in patients with macular impairments such as ERM [11, 12]. However others have reported good visual outcomes with implantation of multifocal IOL in patients with age-related macular degeneration and diabetic retinopathy [12]. One population-based prevalence study on 4439 subjects with an age ≥ 40 years has shown a 10-year incidence of ERM of 8.4% [13]. The cumulative incidence of ERM after cataract surgery has been reported to be 12–17% depending on the series and the time period studied [14, 15].

The purpose of this study is to analyze and compare the visual and anatomical results of PPV for ERM in previously operated eyes of crystalline surgery with implantation of a single diffractive trifocal IOL model.

## Methods

We performed an institutional retrospective cohort study of patients from 5 centers of Clinica Baviera, an AIER Eye Hospital Group Company Ltd. division in Spain. The study was approved by the Institutional Review Board and the Research and Development Department before recruitment was initiated and followed the stipulations of the Declaration of Helsinki. All the patients signed an informed consent document for their data to be retrieved from the electronic medical records. The data recorded included uncorrected distance visual acuity (UDVA), corrected distance visual acuity (CDVA), uncorrected near visual acuity (UNVA), refraction, intraocular pressure, refractive adjustment after lens surgery (bioptics), dye used for ERM and ILM removal, CMT and state of the retinal layers, especially the ellipsoid zone (photoreceptor inner/outer segments [IS/OS]) which was analyzed using spectral domain optical coherence tomography (SD-OCT). We also recorded argon laser photocoagulation data prior to PPV, Nd-YAG laser capsulotomy data, and surgical and postoperative complications. Clinical data were recorded at 3 time points in the clinical history: 3 months after lensectomy, prior to PPV and 12 months after PPV. The 3 SD-OCT platforms used were OCT 2000 (Topcon, Tokyo, Japan), Cirrus HD-OCT (Zeiss, Oberkochen, Germany), and Spectralis (Heidleberg Engineering, Heidelberg, Germany). The lensectomies were performed by 7 experienced cataract surgeons, who recorded no complications during or after the procedure, and the IOL implanted in all cases was the Finevision MicroF® (PhysIOL, Liege, Belgium).

The PPVs were performed by 5 very experienced retinal surgeons using a Constellation Vision System platform (Alcon, Fort Worth Texas, USA) The indication for PPV was determined according to our center’s clinical protocol: metamorphopsia of the eye with ERM, worsened CDVA in more than 1 Snellen line or 5 ETDRS letters, and no clear alternative etiology..The dyes used for removal were Membrane Blue Dual (DORC, Zuidland, The Netherlands), Brilliant Blue (ILM-Blue, DORC, Zuidland, The Netherlands) and Trypan Blue (Membrane Blue, DORC, Zuidland The Netherlands). The PPV calibers used were 23G and 25G.We included 20 eyes from 20 patients who had developed ERM after lensectomy with implantation of a Physiol MicroF® IOL. In all 20 cases, we recorded a posterior vitreous detachment before the PPV. The ERM stage in all cases was 1B or 1C of group 1 according to the classification proposed by Hwang and Sohn in 2012 [16]. There were no cases with pseudohole-type ERM (group 2). The patients underwent PPV with ERM between January 2015 and September 2018. Minimum follow-up was 12 months. Exclusion criteria were intra- or postoperative complications, glaucoma, retinal detachment, recent retinal vascular occlusion syndromes, or any optic nerve impairments before or after lensectomy.

The primary outcome measure was mean change in visual acuity produced by ERM and after PPV in pseudophakic eyes with a trifocal IOL. The secondary outcome measures were change in mean CMT, recovery of the ellipsoid zone after PPV in the SD-OCT which was defined by an image of a continuous hyperreflective line corresponding to the IS/OS junction, which was judged as an intact IS/OS junction (total recovery); when the image showed cysts in the outer layer and a discontinuous IS/OS junction, it was therefore labeled as a disrupted IS/OS junction (no/partial recovery) as defined by Inoue and Morita in 2011 [6]. Other secondary outcomes was CDVA prior to PPV, with measures assessed by comparing 2 groups (one with ≥0.2 logMAR and the other with < 0.2 logMAR), and the shift in spherical equivalent (SE).

### Statistical analysis

Time series were analyzed using traditional ANOVA, when all assumptions were met, or robust ANOVA, when some of the assumptions were violated. Independent samples were compared using the *t* test or the Yuen-Welch test depending on the distribution of the variables. Finally we applied regression methods to measure correlation between numeric variables [17]. All calculations were performed with R version 3.5.3 of 2019 (R Foundation for Statistical Computing, Vienna, Austria).

## Results

We recorded data on 20 eyes from 20 patients (12 males [60%] and 8 females [40%], 13 right eyes [65%] and 7 left eyes [35%]), with a mean age of 63 ± 3 years (range, 54 to 77 years). The mean time between lensectomy and PPV was 607 ± 274 days (range 92 to 1680 days). Follow-up was longer than 12 months after the PPV in all cases with a mean of 447 ± 394 days (range 341 to 911 days). All demographic data are listed in Table 1. No complications were recorded during the PPV procedures, with no visualization difficulties nor increased length of surgery reported by any surgeon.

**Table 1:** Demographics

Patients	20
Age (Min to Max; Mean (±SD))	54 to 77; 63 (±3)
**Sex, No. (%)**	
Male	12 (60%)
Female	8 (40%)
**Eye, No. (%)**	
RE	13 (65%)
LE	7 (35%)
**Stain, No. (%)**	
TRYPAN BLUE	2 (10%)
BRILLIANT BLUE	1 (5%)
DUAL BLUE	17 (85%)
**Caliber, No. (%)**	
23 g	19 (95%)
25 g	1 (5%)
**Bioptics, No. (%)**	
No	18 (90%)
Yes	2 (10%)
**YAG, No. (%)**	
No	19 (95%)
Yes	1 (5%)

Note: Stain = type of stain used for epiretinal membrane peeling; YAG = Neodymium-YAG capsulotomy

### Visual acuity results

**Fig. 1:**
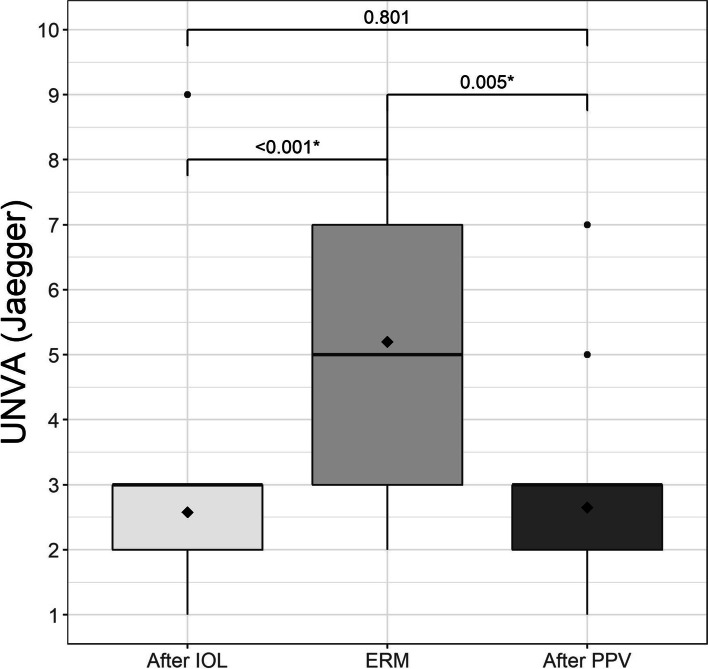
Mean uncorrected distance visual acuity (UDVA) in logMAR after implantation of the trifocal intraocular lens (IOL), when the epiretinal membrane (ERM) appeared, and after pars plana vitrectomy (PPV)

**Fig. 2:**
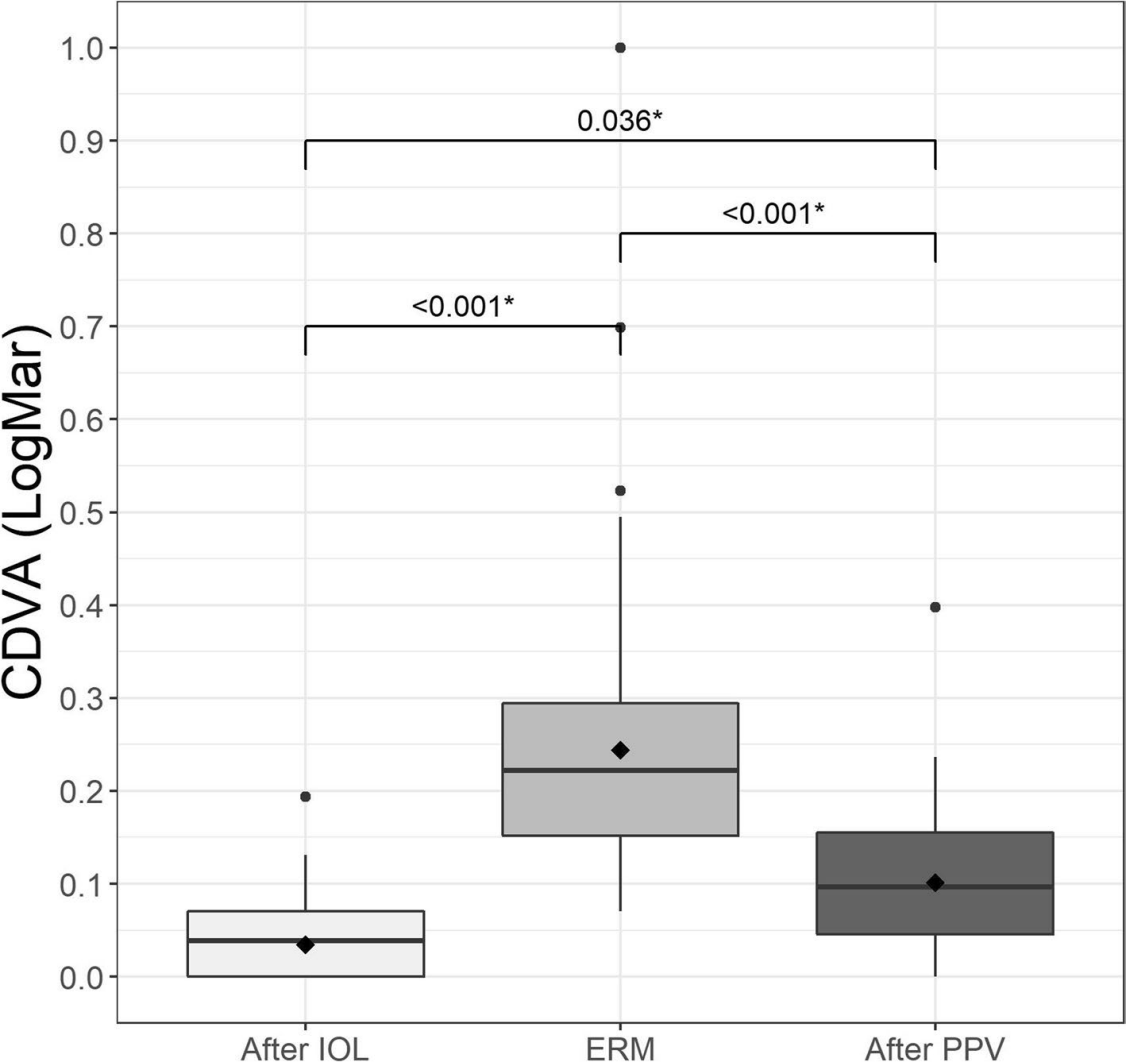
Mean corrected distance visual acuity (CDVA) in logMAR after implantation of the trifocal intraocular lens (IOL), when the epiretinal membrane (ERM) appeared, and after pars plana vitrectomy (PPV)

**Fig. 3:**
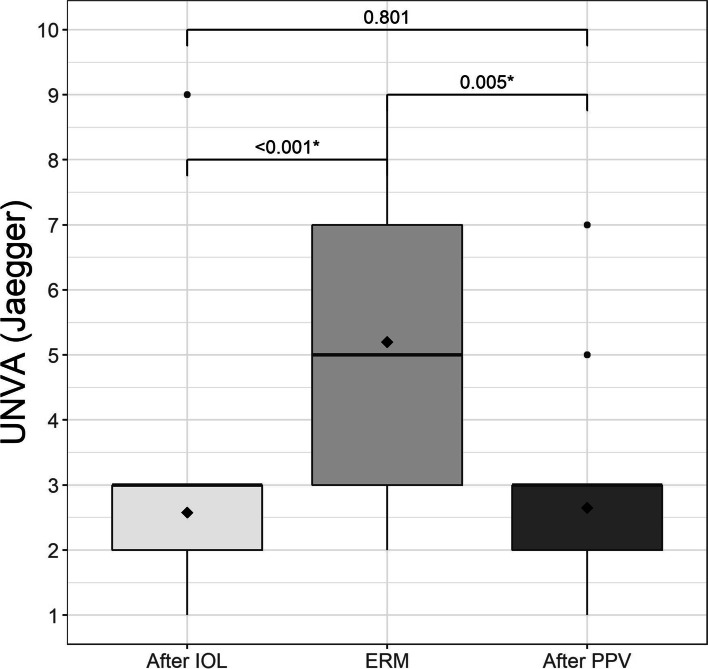
Mean uncorrected near visual acuity (UNVA) in logMAR after implantation of the trifocal intraocular lens (IOL), when the epiretinal membrane (ERM) appeared, and after pars plana vitrectomy (PPV)

**Fig. 4:**
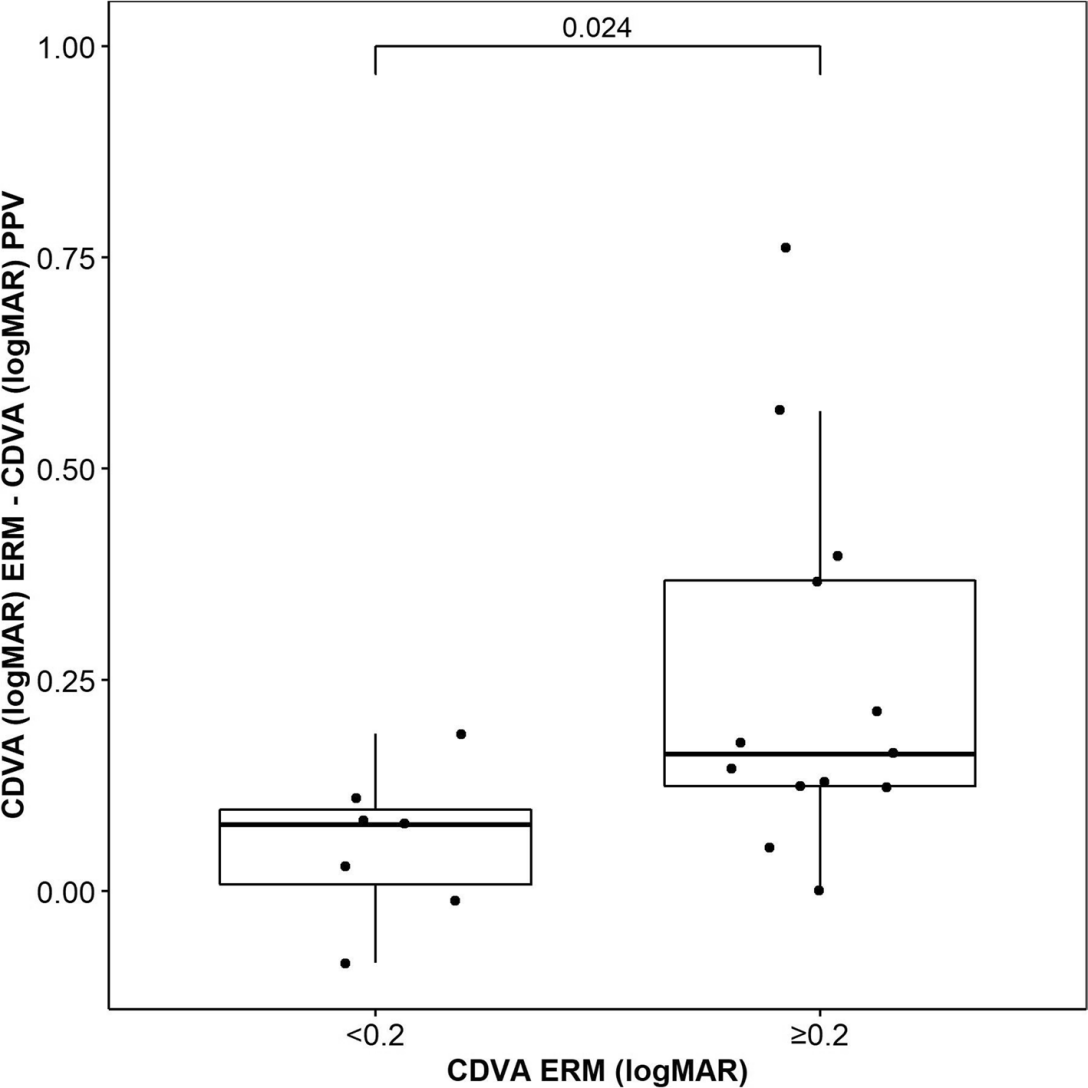
Corrected distance visual acuity (CDVA) in logMAR after pars plana vitrectomy (PPV) vs CDVA when the epiretinal membrane (ERM) appeared (≥0.2 logMAR or < 0.2 logMAR)

Three months after lensectomy, the mean UDVA Snellen was 0.08 ± 0.03 logMAR, which decreased to 0.35 ± 0.13 logMAR at diagnosis of ERM, and 0.19 ± 0.09 logMAR at 12 months after surgery (Fig. 1). CDVA was 0.03 ± 0.03 logMAR after lensectomy and 0.23 ± 0.10 logMAR at diagnosis of ERM, improving to 0.10 ± 0.04 logMAR 6 months after PPV (Fig. 2), with no values greater than 0.39 logMAR. These changes were statistically significant (Table 2). The mean UNVA 3 months after multifocal lensectomy was Jaeger 2.62 ± 0.51. UNVA was worse before PPV Jaeger 5.46 ± 1.67, although it improved to Jaeger 2.69 ± 0.84 at 12 months after PPV (Table 2). These changes were statistically significant (Fig. 3). The mean UNVA 12 months after PPV was Jaeger 2.56 ± 0.51 in eyes with complete recovery of the ellipsoid zone, compared with eyes with partial or no recovery, in which it was Jaeger 4.29 ± 1.01 (*p* = 0.009). A comparison of visual results in 2 groups of CDVA before PPV, one with logMAR< 0.2 and the other with logMAR ≥0.2 revealed statistically significant differences (*p* = 0.024) (Fig. 4).

**Table 2:** Visual Results

Variable	N	After IOL	ERM	After PPV	pValue
Spherical equivalent (D)	20	−0.18(±0.38)	0.02 (±0.40)	−0.18 (±0.47)	0.140*
CDVA (LogMar)	20	0.03 (±0.03)	0.23 (±0.10)	0.10 (±0.04)	< 0.001†
UDVA (LogMar)	20	0.08 (±0.03)	0.35 (±0.13)	0.19 (±0.09)	< 0.001†
UNVA (Jaeger)	20	2.62 (±0.51)	5.46 (±1.67)	2.69 (±0.84)	< 0.001†
OCT	20	NA	380.15(±60.50)	313.70(±36.98)	< 0.001*

Note: N = sample; IOL = intraocular lens; ERM = epiretinal membrane; PPV = pars plana vitrectomy; CDVA = corrected distance visual acuity in logMAR; UDVA = uncorrected distance visual acuity results in logMAR; UNVA = uncorrected near visual acuity results in Jaeger; CMT = central macular thickness in μm

^*^Repeated measures ANOVA

^†^Robust repeated measures ANOVA due to outliers

### SD-OCT based anatomic results

**Fig. 5:**
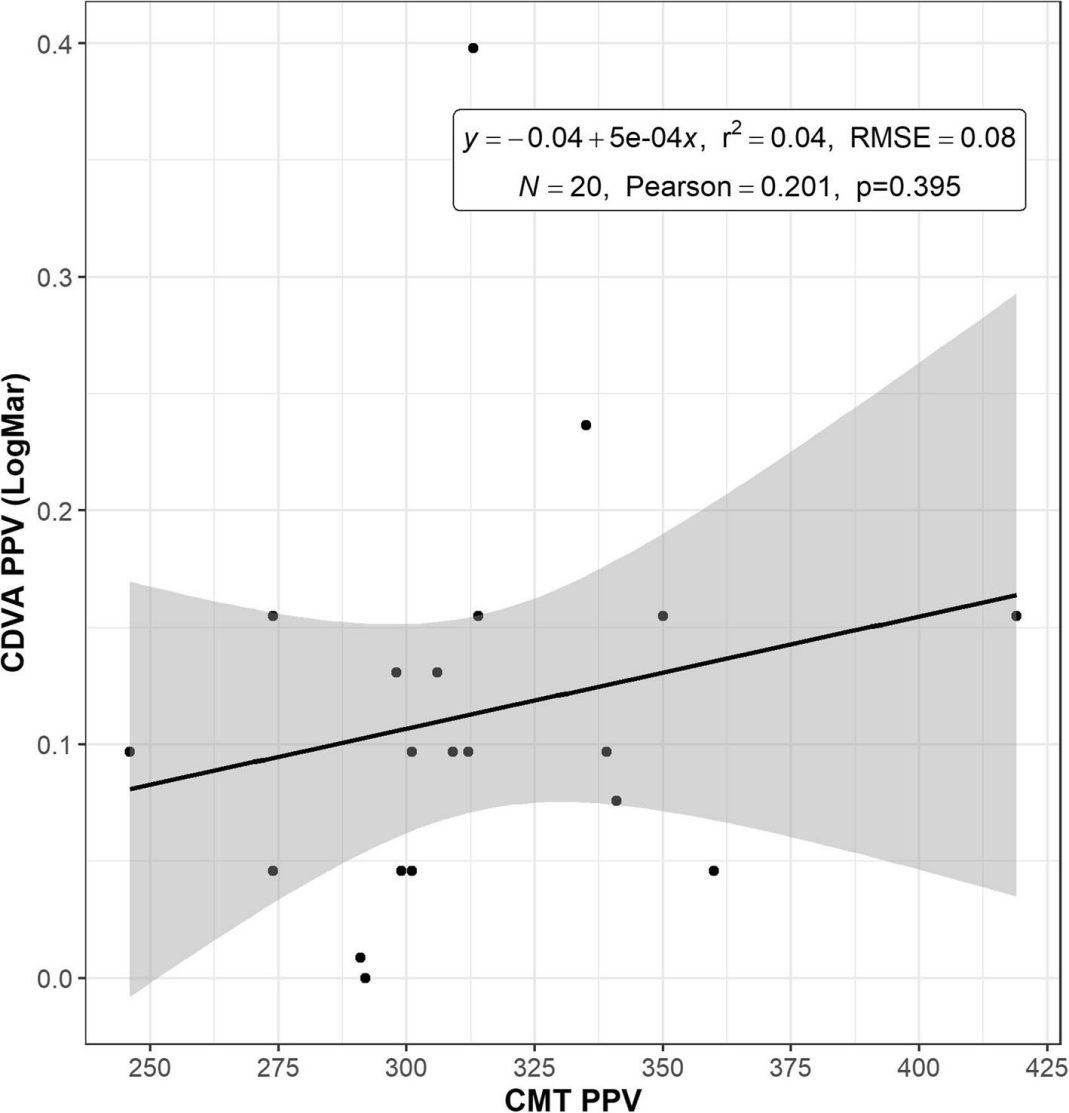
Correlation between mean corrected distance visual acuity (CDVA) in logMAR and mean central macular thickness (CMT) registered with spectral domain optical coherence tomography (OCT) in microns after pars plana vitrectomy (PPV)

Mean CMT at diagnosis of ERM was 380.15 ± 60.50 μm (range, 312 to 501 μm). Six months after PPV, the mean CMT had fallen to 313.70 ± 36.98 μm (range, 274 to 419 μm), and although the difference was statistically significant (*p* = 0.00083, *t* test) (Table 2), there was no strong correlation after application of the Pearson and ordinary least squares methods (Fig. 5). We observed a correlation between mean CDVA 12 months after vitrectomy and recovery of the ellipsoid zone, which was 0.07 ± 0.03 logMAR in patients with complete recovery, and 0.14 ± 0.06 logMAR in those with an incomplete recovery (*p* = 0.022) (Table 3).

**Table 3:** Visual Results Comparing Recovery of the External Retinal Layers (ERL)

IS-OS recovery	N	Mean (±)	Range (Min/Max)
Yes	11	0.07 (±0.03)	0.00/0.15
No	9	0.14 (±0.06)	0.08/0.40

Note: N = sample; IS-OS = ellipsoid zone; yes = complete recovery of external retinal layers (ERL); no = incomplete recovery of ERL

Due to outliers trimmed means are shown. Yuen test for trimmed means, *p* = 0.022

### PPV technique-related results and complications

The dyes used were Membrane Blue Dual in 17 eyes (85%) and Brilliant Blue in 2 eyes (10%) and Trypan Blue in 1 eye (5%). ILM peeling was not confirmed in 2 of the 20 cases included, which were those in which Trypan Blue was used as a dye. As for PPV calibers we recorded 23G in 19 eyes (95%) and 25G in 1 eye (5%). No recurrence of ERM was observed during follow-up. We did not observe any correlation between CDVA after PPV and the time lapse between lensectomy and PPV (*p* = 0.92). The correlation between the CDVA and the dye or the caliber of PPV used for the removal of ERM could not be established owing to insufficient number of cases.

A bioptics procedure by photorefractive keratectomy was performed 3 months after lensectomy in 2 eyes (10%) and no refractive adjustment was made after vitrectomy. Nd-YAG laser capsulotomy was necessary in 1 eye (5%) 24 months after lensectomy and 6 months before PPV. No cases of tilting or decentration of the lens were observed, during or after the PPV.

The only complication we recorded after PPV (5%) was a case of cystoid macular edema in a diabetic patient with no signs of diabetic retinopathy who responded well to topical nonsteroidal anti-inflammatory drugs and corticosteroids (final CDVA, 0.07 logMAR).

### Spherical equivalent change results

**Fig. 6:**
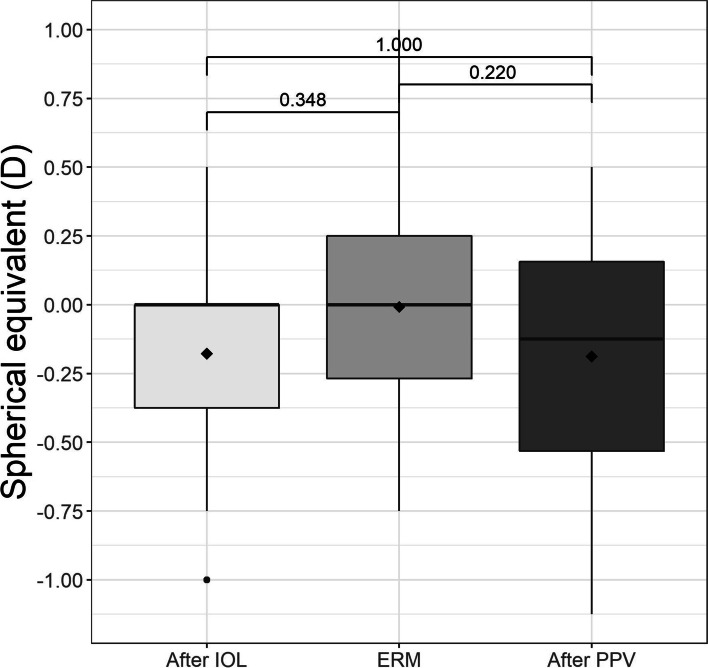
Spherical equivalent (SE) in diopters (D) after implantation of the trifocal intraocular lens (IOL), when the epiretinal membrane (ERM) appeared and after pars plana vitrectomy (PPV)

Comparison of the SE 3 months after lensectomy with the SE at diagnosis of ERM showed that the mean value had changed from − 0.18 ± 0.38 D to 0.02 ± 0.40 D; this difference was not significant (*p* = 0.14). When the mean SE after lensectomy is compared with the SE 12 months after PPV (− 0.18 ± 0.21 D), the difference continued to be nonsignificant (*p* = 0.98) (Table 2) **(**Fig. 6**)**. We also compared the mean change in SE after lensectomy with the SE at the last visit 12 months after PPV in two groups, one with sutured sclerotomies and the other without, with no observed statistically significant difference (*p* = 0.256).

## Discussion

Our results are similar in terms of mean gain in CDVA to those seen in patients with monofocal IOL implants who underwent PPV for ERM [18–21], thus mitigating one of the main concerns with respect to implantation of multifocal IOLs, which is the rationale of this study. This is consistent with the outcomes observed for multifocal IOL implants in eyes with other macular diseases, such as aged-related macular degeneration and diabetic retinopathy [12]. Our observation seems to be more associated with macular SD-OCT findings in the ellipsoid zone (IS/OS junction) recovery, as observed and discussed by Inoue and Morita [6, 22]. The mean UNVA results also improved significantly after the decrease recorded at diagnosis of ERM, which is also associated in our study with complete recovery of the ellipsoid zone. The observed decrease was based on comparison with eyes for which no recovery was recorded or recovery was only partial, although an acceptable UNVA was maintained in some of these eyes.

None of the surgeons reported any perioperative difficulty related to the design of the diffractive rings or lens, despite the data published by Yoshino and Inoue in 2010 [23], who reported visualization difficulties during PPV for ERM. However, it is important to note that the IOL model in that case was different (ZM900®; Abbott Medical Optics, Johnson and Johnson Vision, Santa Ana California, USA).

We recorded the number of Nd:YAG laser capsulotomies after lensectomy or either after PPV as an objective measure of posterior capsule opacification (PCO) rate. This procedure was necessary in only one eye (5%) 24 months after lensectomy, an incidence similar to the 9% observed in 1830 eyes with this trifocal lens model after a minimum follow up of 12 months by Bilbao-Calabuig R et al. in 2016 [24]. They compared this group with another of 1015 eyes with an AT Lisa tri 839MP (Carl Zeiss Meditec, Jena, Germany) implanted at the same period which showed an incidence of ND:YAG capsulotomy of 23%. That difference is explained by the design of both IOL platforms, specifically the differences in the flexibility between the tetraloop design (FineVision MicroF IOL) and the plate-haptic design (AT Lisa tri 839MP), as well as differences in the haptic–optic junction. This could also explain the absence of tilting or decentration of this lens model after the PPV.

Consistent with reports from other authors [18–21], the best mean CDVA results were obtained in eyes with better visual acuity before vitrectomy, with a *p* value of 0.024 between patients with a CDVA logMAR < 0.2 and those with a CDVA logMAR ≥0.2. This finding supports the indication of this procedure for symptomatic eyes diagnosed with ERM and good CDVA.

We did not observe any case of retinal detachment even though this complication has been reported after 1 year of follow-up in a large series of eyes with monofocal lenses (362 eyes) undergoing PPV for ERM (2.5%) by Guillaubey and Malvitte [25]. Inclusion of more cases in our study may have revealed cases of retinal detachment.

No significant myopic shift was observed in our study when SE after lensectomy was compared with SE after PPV, even when comparing eyes with or without sutured sclerotomies, thus explaining why no refractive adjustment after vitrectomy was required. In a similar study of 28 pseudophakic eyes with a monofocal IOL, Hamoudi and Kofod in 2013 observed a clearly significant myopic shift after PPV, although the follow–up period was 8.5 months [26]. In another review article, Hamoudi and La Cour reported different shifts in SE, even though other conditions and techniques were included [27].

In their pilot study, Patel et al. implanted a bifocal IOL (AcrySof IQ ReSTOR® SN6AD1; Alcon Laboratories, Inc., Fort Worth, TX, USA) during phacoemulsification combined with PPV in 6 eyes with cataract and ERM. The authors reported good visual and anatomical outcomes after only 3 months, despite using indocyanine green to stain [28]. Even though visual results with this trifocal IOL model are good, we do not consider its implantation when performing a combined procedure of phacoemulsification and PPV for ERM that could potentially work but involves a high risk of intolerance and may require explantation of the IOL. A safer option could be to perform the PPV for ERM and later on the phacoemulsification with a trifocal IOL implantation, once the macular recovery is complete, but a prospective study is needed to confirm it.

To our knowledge this is the largest study to date which examines visual outcomes for this procedure in eyes with this trifocal IOL model. Despite the relatively small sample size of 20 eyes, our results suggest that this trifocal IOL does not affect visual outcomes. One strength of the study is the long follow-up period (> 12 months) for all of the eyes included. Our study is limited by its retrospective design and the use of 3 different SD-OCT devices (because of potential interference with the CMT which made it impossible to compare the ERM 1B and 1C Hwang’s stages with VA results). We were not able to perform an adjustment of error between these measurements because the OCT images that were incorporated into the medical records did not allow it. Finally this study is also limited by the absence of measurements of contrast sensitivity and metamorphopsia due to the lack of such tests included in the complementary test protocol for PPV in our center, and the fact that surgery and follow-up involved more than 1 surgeon.

## Conclusions

PPV for ERM peeling in eyes with this trifocal diffractive IOL seems to be safe and effective, with good visual results not only for distance, but also for near visual acuity. Larger scale and prospective studies are needed to confirm our observations.

## Data Availability

The datasets analyzed during the current study are available from the corresponding author on reasonable request.

## Abbreviations

CDVA: Corrected distance visual acuity
CMT: Central macular thickness
ERM: Epiretinal membrane
ILM: Internal limiting membrane
IOL: Intraocular lens
IS/OS: Photoreceptor inner segment/outer segments junction (ellipsoid zone)
Nd-YAG: Neodymium-doped yttrium aluminum garnet
PPV: Pars plana vitrectomy
SD-OCT: Spectral domain optical coherence tomography
SE: Spherical equivalent
VA: Visual acuity
UDVA: Uncorrected distance visual acuity
UNVA: Uncorrected near visual acuity
